# 

**Published:** 1856-03

**Authors:** 


### CONTENTS.

ORIGINAL COMMUNICATIONS— PAGE.

Valedictory Address before the Aesculapian Society, at Charleston, Ill., Nov. 28th, 1855. By Thos. D. Washburn, M.D., of Lawrenceville, Ill., 103

Injury to the Perineum puncture of the Bladder above the Pubes. Reported by Dr. George M. Fox, Brush Hill, Ill., ....115

Some Remarks in Reference to the Necessity of Early and Free Venesection in the Treatment of Meningitis—with Cases. By J. W. McKenney, M.D., New Albany, Ill., Jan. 1856, ....118

Double and Compound Fracture of the Thigh. Fatal after 74 days. By David Prince, M.D., Jacksonville, Ill., ....125

SELECTIONS—

Abdominal Dropsy, ....127

Clinic of Dr. Biddle. ....130

BOOK NOTICES, ....133

EDITORIAL, ....138

